# Spirometry to increase smoking cessation rate: A systematic review

**DOI:** 10.18332/tid/106090

**Published:** 2019-04-17

**Authors:** Elisabeth Westerdahl, Kjell Ola Engman, Mats Arne, Matz Larsson

**Affiliations:** 1 Centre for Assessment of Medical Technology in Örebro, Region Örebro County, Örebro, Sweden; 2 Department of Physiotherapy, University Health Care Research Center, Faculty of Medicine and Health, Örebro University, Örebro, Sweden; 3 Sörmland County Council, Medical Advisory Committee, Nyköping, Sweden; 4 Centre for Clinical Research, Region Värmland, Karlstad, Sweden; 5 Department of Medical Sciences, Respiratory, Allergy and Sleep Research, Uppsala University, Uppsala, Sweden; 6 Clinical Health Promotion Centre, Lund University, Lund, Sweden; 7 The Heart, Lung and Physiology Clinic, Örebro University Hospital, Örebro, Sweden; 8 School of Medical Sciences, Faculty of Medicine and Health, Örebro University, Örebro, Sweden

**Keywords:** smoking cessation, prevention, spirometry

## Abstract

**INTRODUCTION:**

Addressing tobacco use is an important issue in general health care. In order to improve smoking cessation advice, spirometry values can be displayed to the smoker to demonstrate possible lung function impairment. The estimate of so-called lung age may show a decrease in lung function associated with smoking. It has been suggested that performing spirometry on patients who smoke but are asymptomatic can be a useful way to show the adverse effects of smoking. The aim of this systematic review was to determine if providing spirometry results in combination with smoking cessation counselling can increase smoking cessation rates compared to what is achieved through counselling alone.

**METHODS:**

In this systematic review, we included randomized controlled trials (RCTs) evaluating smoking cessation interventions for adult smokers. The systematic search was performed in PubMed, Medline, Cochrane Library, Cinahl, Embase, Amed and PsycInfo.

**RESULTS:**

The literature search resulted in 946 studies, which, after reading by two independent reviewers, were reduced to seven trials that matched the inclusion criteria. Two RCTs showed significant improvement in smoking cessation when giving patients feedback on spirometry results in combination with smoking cessation counselling, compared to patients who received only smoking cessation counselling. In both studies, the spirometry results were expressed as lung age. In the other five studies no difference was found. Five further published study protocols for ongoing RCT studies in the field have been found, and therefore this systematic overview will likely need to be updated within a few years.

**CONCLUSIONS:**

Few studies have been undertaken to examine the efficacy of spirometry in increasing smoking quit rates. Studies conducted to date have shown mixed results, and there is currently limited evidence in the literature that smoking cessation counselling that includes feedback from spirometry and a demonstration of lung age promotes quit rates.

## INTRODUCTION

All smoking, including occasional smoking and even smoking in small amounts, is associated with a sharply elevated risk of disease, reduced quality of life and premature death^1^. Many healthcare systems offer expert counselling to patients who smoke. Various types of measures, such as simple advice, counselling in person and proactive telephone counselling have proven to be effective. There is, however, no consensus on how counselling should be conducted to achieve the best effect.

Ceasing smoking is often highly challenging for long-term smokers, and both motivation and perseverance are required. To achieve success, smokers must first and foremost decide that they want to quit smoking. There are many methods to facilitate cessation, including nicotine replacement products, prescription of medications that reduce the craving for tobacco, and participation in quit-smoking groups.

Smoking is associated with an increased risk of about 60 disease diagnoses and is a predominant cause of many common diseases including cancer, cardiovascular disease and chronic obstructive pulmonary disease (COPD)^1^. In advanced stages with low FEV1 (forced expiratory volume in one second), COPD is a serious condition associated with shortness of breath, limited physical capacity, decreased quality of life and risk of premature death. Spirometry provides valuable information regarding the presence of COPD and the degree of severity. Smoking cessation is the only intervention that can slow disease progression and decrease worsening of pulmonary function over time^2^.

Simple dynamic spirometry is a useful healthcare tool to measure pulmonary function in smokers and help motivate them to quit smoking. If spirometry shows signs of airway obstruction as demonstrated by a decrease in forced expiratory volume in one second (FEV1) or FEV1/forced vital capacity (FVC), the findings can be presented to the patient as evidence of a decrease in lung function. The ‘lung age’ of the smoker can also be calculated, and development of airway obstruction can be demonstrated graphically in relation to age^3^.

Lung age is defined as the average age of a person with the same FEV1 as that measured for the patient. This lung age can then be compared with the chronological age of the individual. Equations for the determination of lung function and lung age have been developed from reference values and linear regression equations^4^. The predictive formula uses the patient’s gender, height and measured FEV1 to determine the age for which the predictive FEV1 value is 100%^4^.

Smoking cessation is an area of high priority within healthcare. There is evidence that advice and guidance from healthcare personnel concerning smoking cessation are effective interventions that help people quit smoking. Low-intensity advisory interventions are also effective, but no single advisory technique has proven to be superior to any other^5^.

Conducting dynamic spirometry to demonstrate lung age in patients who smoke, even when asymptomatic, has been proposed as a useful intervention to motivate patients to quit smoking. However, it remains unclear whether the addition of spirometry to smoking cessation counselling is actually helpful in increasing smoking quit rates. The purpose of this systematic review of the literature is to clarify whether feedback from spirometry results has an additive effect in helping adult smokers to cease smoking, compared with smoking cessation counselling alone.

## METHODS

### Inclusion criteria

This systematic review includes randomized controlled studies that assess healthcare interventions for smoking cessation in adult (>18 years) smokers who are offered smoking cessation counselling. Smoking refers to daily smoking, regardless of number of cigarettes smoked. The intervention includes smoking cessation counselling with spirometry including feedback of the results (e.g. FEV1, FEV1/FVC or lung age) to improve motivation to quit smoking. Subjects in the control group were not informed of their spirometry results. The percentage of smokers who quit smoking following the intervention was assessed. Selection criteria for inclusion in the studies were:

- Study design: randomized controlled trials (RCTs).
- Study participants: adults who smoke and who participate in smoking cessation, respiratory disease screening, or health monitoring programmes.
- Intervention: all interventions in which spirometry results are used to increase motivation to quit smoking. The spirometry results can be presented to the patient as a single component or as a complement to other interventions such as counselling. The control group receives: 1) all components except spirometry results, or 2) no intervention at all.
- Outcome measure: percentage smoking cessation, measured at least one month following start of intervention.
- Publication date: no limitations regarding year of publication.

### Exclusion criteria

- Narrative review article
- Congress abstract
- Language other than English or a Scandinavian language

### Literature search

A systematic search of the literature was conducted on 27 March 2017 by the librarian at Örebro University in the following databases: PubMed, Medline, Cochrane Library, Cinahl, Embase, Amed and PsycInfo. The search strategy is presented in Appendix 1.

The following keywords were formulated in PubMed and adapted to the other databases. The search was based on the following search string: ((smok*[Title/Abstract]) OR (‘smoking cessation’[MeSH Terms] OR ‘smoking cessation’[All Fields]) AND (intervention[Title/Abstract] OR program*[Title/Abstract] OR support[Title/Abstract] OR cessation[Title/Abstract] OR stop[Title/Abstract]) AND ((‘Respiratory Function Tests’ OR fev1) OR (‘lung age’) OR (forced expiratory volume) OR ‘Respiratory Function Tests’[Mesh]) OR ((copd-6 OR copd6) OR Vitalograph) OR spirometr*) OR spirometry[MeSH Terms]).

Limitations: Clinical trials and systematic reviews were included. Duplicates were eliminated by the librarian in charge of the search.

The PROSPERO database^6^ was searched on 9 October 2017 for ongoing systematic reviews using the keywords ‘smoking cessation’ AND ‘spirometry’; seven study protocols were found, but none was relevant to our research question. At Clinicaltrials.gov^7^, five study protocols for RCTs were found related to the use of spirometry as a strategy for smoking cessation.

### Data extraction

Two independent authors reviewed the search results by individually reading titles and abstracts. Articles were selected when either of the two authors deemed it appropriate, after which these articles were read in full and ultimately included if they were still considered to meet the inclusion criteria. If the same study investigated multiple intervention groups with non-relevant interventions, such as nicotine chewing gum or treatment with medications, the study was still included but the results for these groups were omitted in the compilation of relevant results.

### Assessment of methodological quality

The authors conducted quality assessment individually according to the SBU template for quality assessment of randomized studies^8^, after which disagreements were resolved by consensus.

## RESULTS

**Figure 1:**
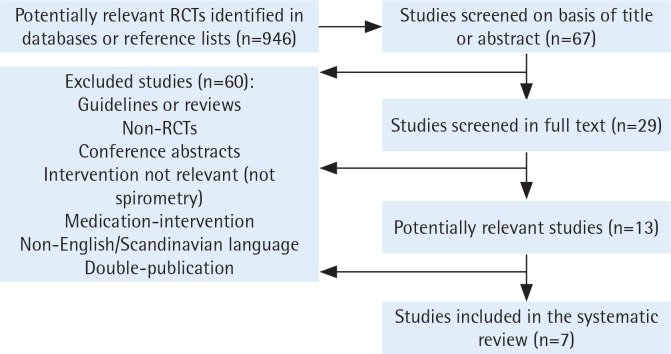
Flowchart for inclusion of studies in the systematic review

Seven randomized controlled studies (total study population n=1935) fulfilled the inclusion criteria for this systematic review (Figure 1, Table 1). We excluded nine other studies since the intervention did not consist of feedback from spirometry, or they were not RCTs.

**Table 1:** Summary of included studies (n=7 )

Authors, Year, Country	Population/participants	Intervention group/Control group (n = patients analysed)	Primary outcome measurement	Results
Drummond et al.^15^ (2014) USA	Residents of Baltimore (n=45) who were ≥18 years of age and had a history of injecting drugs were invited. Eligibility requirements included current cigarette smoking (at least 100 cigarettes in their lifetime as well as reporting any cigarette smoking in the last month), no current involvement in a smoking cessation programme, no current use of nicotine replacement therapy or other smoking cessation pharmacological treatments, and the ability to perform spirometry.	Patients were randomized to one of four groups (only Lung age group and CG presented here). IG (Lung age intervention) (n=20). Spirometry results were reviewed in the context of lung age. Visual graphs were used to explain how the lung function normally reduces with age and that smoking can damage lungs in a manner similar to more rapid aging. Written report included their chronological age and lung age. The threshold to define abnormal was lung age exceeding chronological age. CG (Usual care) (n=25). Spirometry results of their lung function were reported as a percentage of predicted values, communicated in a standardized written format.	Six-month biologically-confirmed smoking cessation (self-report of non-smoking in the last seven days combined with negative CO and serum cotinine). One baseline visit and six follow-up visits over six months.	The six-month biologically-confirmed smoking cessation rate was 4% for usual care and 0% for the lung age intervention group. No effect of using spirometric lung age as tool to change smoking behaviour in this population was found when compared to usual care.
Ojedokun et al.^14^ (2013) Ireland	Patients (n=290) undergoing routine consultations at two rural and three urban general practices in Ireland, regardless of the reasons, on a given day in primary care. Non-smokers were excluded. Exclusion criteria: unavailability for follow-up, enrolment in another smoking cessation research study, current use of smoking cessation pharmacotherapy, use of domiciliary oxygen, history of major lung disease and cognitive dysfunction.	IG (n=140): In addition to standardized personalized brief smoking cessation advice, participants additionally had their lung age assessed using the desktop Vitalograph lung age meter (portable desktop device). Lung age results was explained, recorded on an advice slip and given to these patients. It estimates the lung age to help illustrate the impact of smoking on the subjects' lungs based on the age, height, gender and FEV1. CG (n= 150) All patients received standardized personalized brief smoking cessation advice including an offer of cessation support in the form of pharmacotherapy or a follow-up review as appropriate and also the standard patient information leaflet.	Proportion of patients abstinent from smoking for one month after intervention (selfreported).	Self-reported quit rates at 4 weeks in the intervention and control arms respectively were 22% and 12% (p=0.01). Our principal finding is that, in addition to brief cessation support during routine consultations, providing lung age bio-feedback to smokers along with pharmacotherapy significantly increases the proportion who quit within a month.
Kaminsky et al.^13^ (2011) USA	Participants (n=67) were current smokers referred to the pulmonary function test laboratory by their physician for shortness of breath, abnormal chest X-ray, cough, or preoperative evaluation. The trial was explained as a study of the smoking habits of patients having pulmonary function tests. The true nature of the study, to determine the effects of the intervention on quit attempt rate, was not revealed at that time.	IG (n=34): The technologist completed the lung function testing and helped the participant find his/her lung age on a graph drawn according to Fletcher and Peto and followed a standardized script to share lung function results with participants in order to enhance their motivation to quit (15 minutes) CG (n=33): Received an information sheet on smoking cessation resources in the community as recommended by current guidelines (1 minute).	Quit attempt rate at 1 month after intervention.	The incidence of one or more quit attempts at 1 month was n=8 (24%) control vs n=11 (32%) intervention, with no significant difference between groups.
Kotz et al.^10^ (2009) The Netherlands	Current smokers (n=296) from the general population (recruited through newspapers, flyers) with previously undiagnosed mild-to-moderate airflow limitation by means of spirometry. Eligibility was assessed during an initial telephone interview. Inclusion criteria were: smoking history of ≥ 10 pack-years, reading and speaking Dutch and reporting at least one of the symptoms (cough, sputum production or shortness of breath). Exclusion criteria: prior respiratory diagnosis, or having undergone spirometry during the preceding 12 months.	IG (n=116) Exp: Medium-intensity confrontational counselling discussing the spirometry results and confronting the consequences of smoking: diagnosis (COPD) delivered by a respiratory nurse combined with nortriptyline for smoking cessation (4 sessions á 40 min). Spirometry was performed on a Vitalograph^®^ 2120 (Vitalograph Ltd, Buckingham, UK). CG 1 (n=112): Medium-intensity health education and promotion delivered by a respiratory nurse combined with nortriptyline for smoking cessation (4 sessions á 40 min). CG 2 (n=68): Low intensity 'care as usual' by the general practitioner.	Prolonged abstinence from smoking from weeks 5 to 52 after the target quit date. Prolonged abstinence was defined as urine cotinine-validated (<50 ng·mL^-1^).	There was no significant difference in prolonged abstinence rates from weeks 5 to 52. Confrontational counselling discussing spirometry results did not increase the prolonged abstinence from smoking rate from weeks 5 to 52 compared with an equally intensive treatment in which participants were not confronted with spirometry.
Parkes et al.^12^ (2008) United Kingdom	Current smokers (n=561) aged over 35 from five general practices. Computerized patient records were searched to identify patients who had been recorded as smokers in the previous 12 months. Exclusion: Patients receiving oxygen, history of lung cancer, tuberculosis, asbestosis, silicosis, bronchiectasis, or pneumonectomy.	IG (n=280): Were given their spirometry results verbally, in the form of 'lung age' with a graphic display after the test and written results by letter within four weeks. CG (n=281): Were not informed of their results, except for a written result as simple FEV1 (absolute values) with no further explanation by letter within four weeks. All participants underwent standard measurements of lung function (FEV1, FVC, FEV1/FVC) with a MicroLab 3500 spirometer (Micro Medical, Chatham, Kent). Both groups were told that their lung function would be measured again after 12 months to see whether it had deteriorated. All were strongly encouraged to give up smoking.	Verified cessation of smoking 12 months after the initial recruitment examination. Smoking cessation at follow-up was initially assessed by measuring carbon monoxide concentrations. Saliva cotinine testing was recorded for assessment of nicotine replacement therapy.	Verified quit rates were 6.4% in the control group and 13.6% in the intervention group (p=0.005). Telling participants their lung age was associated with an absolute reduction of 7% in the smoking rate compared with the CG.
Buffels et al.^11^ (2006) Belgium	Primary care patients (n=221) with a motivation in stage 3 (preparation) or 4 (action) in the scheme of Prochaska and Di Clemente were asked to fix a day to quit smoking, and a follow-up contact was offered. All patients were prescribed nicotine replacement therapy and/or bupropion	IG (n=89): Performed office spirometry and confrontation with their lung function measurement values and their flow/volume curve (normal lung function or airflow limitation defined as a FEV1/FVC <0.7). CG (n=132): No spirometry performed.	Follow up by telephone 6, 12 and 24 months after stop date. Sustained quitters after 2 years were invited to deliver a urine sample for cotinine and creatinine as a control for verification of smoking cessation.	No significant difference between groups regarding success rates at any time point.
Segnan et al.^9^ (1991) Canada	Patients (n=923) who were smokers and free of any life-threatening disease.	Minimal intervention, one face-to-face counselling (n=62). Repeated counselling (RC) in addition to the first counselling, at months 1, 3, 6 and 9 (n=275). RC plus nicotine gum (n=294). RC plus spirometry (in a specialized center). The report form showed an estimate of the 'lung age' of the subject, discussed with the patient by a physician, stressing the need to maintain lung function or not do further damage (n=292).	Biochemically verified smoking-cessation at 12 months after recruitment, sustained for at least three months before the follow-up interview. Selfreported smoking status was validated by determination of urinary cotinine levels.	Smoking cessation rates at 12 months did not significantly differ between groups. Combining repeated counselling with spirometric testing did not result in a significant difference in smoking cessation rates in our study.

The subjects were smokers recruited from the general population^9,10^, primary care^11,12^, or another healthcare institution^13-15^ (Table 1).

The number of participants in the studies varied between 45 and 567. All studies included both men and women. The definition of being a smoker at inclusion in the studies varied and often lacked precision. Two studies were conducted in the US^13,15^, and the remainder were conducted in Canada^9^, Belgium^11^, the UK^12^, the Netherlands^10^ , and Ireland^14^ (Table 1).

The intervention in five of the studies^9,12-15^ involved informing the subjects about their spirometry results (FEV1) through an estimate of their lung age with or without an explanatory graphic display of lung function impairment related to age, while in two studies^10,11^ the patients were provided with their FEV1 values or their FEV1/FVC values either as absolute values or as a percentage of the expected value.

The healthcare personnel who presented the results of the intervention were doctors^9,11,12,14,15^, nurses (respiratory nurse)^10^ or biomedical analysts (pulmonary function technologist)^13^.

The results show that two RCT studies^12,14^ found an improved rate of smoking cessation when smokers were provided with spirometry results in addition to smoking cessation counselling. The follow-up time for these two studies was one month^14^ and one year^12^, respectively. The other five studies showed no statistically significant differences.

The majority of the studies (n=5) were assessed to be at low risk of bias (good-quality study), while two studies were at medium risk of bias (medium-quality study) according to the SBU review template^8^ (Tables 2 and 3). None of the studies reported on health economics analyses or on whether any negative consequences of the intervention occurred.

**Table 2:** Study quality assessment of the included studies — risk of bias

Authors and Year	Selection bias	Performance bias	Detection bias	Attrition bias	Reporting bias	Conflict of interest	Summary
Drummond et al.^15^ (2014)	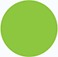	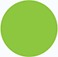	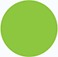	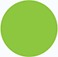	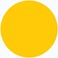	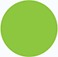	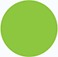
Ojedokun et al.^14^ (2013)	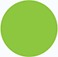	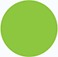	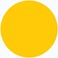	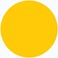	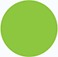	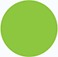	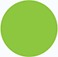
Kaminsky et al.^13^ (2011)	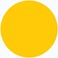	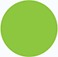	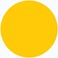	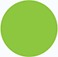	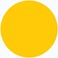	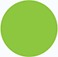	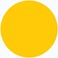
Kotz et al.^10^ (2009)	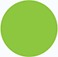	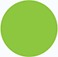	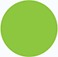	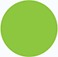	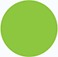	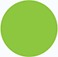	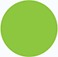
Parkes et al. ^12^ (2008)	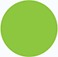	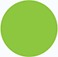	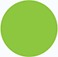	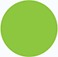	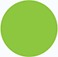	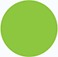	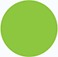
Buffels et al.^11^ (2006)	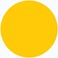	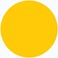	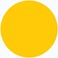	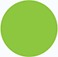	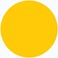	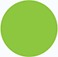	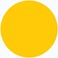
Segnan et al.^9^ (1991)	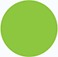	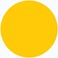	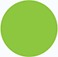	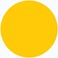	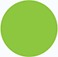	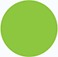	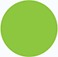

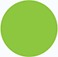
 Low risk of bias 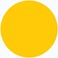 Medium risk of bias

**Table 3:** Summary of direction of effects of spirometry on smoking cessation, study design randomized controlled trials

Authors, Year, Country	Study quality	Sample size	Intervention (in addition to smoking cessation advice)	Spirometry equipment	Compared to	Follow-up (latest)	Effect on smoking cessation
Drummond et al.^15^ (2014) USA	A	20/25 (two more groups not presented here)	Spirometry results communicated to the patient in the context of ‘FEV1/Lung age’ and visual graphs (Fletcher) were used to explain how age and smoking affect lungs (by the primary care provider/general practitioner).	KOKO^®^-pneumotachometers (nSpire Health Inc, Longmont, CO, USA).	Spirometry results (FEV1) reported as a percentage of predicted values (normal/ abnormal) in a standardized written format.	6 months	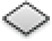
Ojedokun et al.^14^ (2013) Ireland	A	140/150	‘FEV1/Lung age’ results were explained, recorded on an advice slip and given to the patients (by the general practitioner).	Vitalograph lung age meter, COPD-6 (portable desktop device).	Brief smoking cessation advice.	1 month	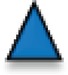 (p=0.01)
Kaminsky et al.^13^ (2011) USA	B	33/34	‘FEV1/Lung age’ was shown to the patient on a graph (Fletcher) and followed a standardized script to share lung function results (15 minutes) (by pulmonary function test technologist).	Pulmonary function test laboratory (details not given).	Information sheet on smoking cessation resources in the community, current guidelines (1 minute).	1 month	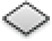
Kotz et al.^10^ (2009) The Netherlands	A	116/68 (one more group not presented here)	Confrontational counselling discussing the spirometry results (FEV1, FEV1/FVC) and confronting of consequences combined with medication (by a respiratory nurse).	Vitalograph^®^ 2120 (Vitalograph Ltd, Buckingham, UK).	Health education and promotion combined with medication.	1 year	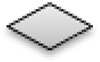
Parkes et al.^12^ (2008) United Kingdom	A	280/281	Spirometry results given verbally, in the form of ‘FEV1/Lung age’ with a graphic display (Fletcher) after the test and written results by letter within four weeks (by general practitioners/principal research doctor).	MicroLab 3500 spirometer (Micro Medical, Chatham, Kent, UK).	Not informed of their spirometry results, except for a written simple FEV1 with no further explanation.	1 year	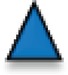 (p=0.005)
Buffels et al.^11^ (2006) Belgium	B	89/132	Confrontation with patients’ lung function measurement values (FEV1/FVC) and their flow/volume curve (normal or airflow limitation) (by a general practitioner).	Office spirometry.	No spirometry performed.	2 year	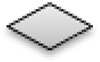
Segnan et al.^9^ (1991) Canada	A	275/292 (two more groups not presented here)	Repeated counselling at months 1, 3, 6 and 9 plus spirometry and an estimate of ‘FEV1/Lung age’ (by a physician).	Spirometry test in a specialized center of the National Health Service.	Repeated counselling at months 1, 3, 6 and 9.	1 year	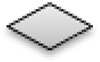

A= low risk of bias, B = moderate risk of bias, C = high risk of bias. Final sample size in each group <50
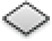

Effect direction: positive outcome
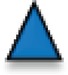

Effect direction: negative outcome
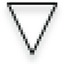

No change/conflicting findings
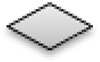

In conjunction with the systematic search we found five published study protocols^16-20^. No additional ongoing studies were found in the PROSPERO database^6^.

## DISCUSSION

This systematic review shows that there is limited scientific support for the theory that providing spirometry results (FEV1 and/or lung age) to adult smokers contributes to a higher rate of smoking cessation. Two from a total of seven studies (RCTs) show significantly improved results when smokers are informed of spirometry results in addition to smoking cessation counselling compared with conventional smoking cessation counselling alone. The two studies were considered to be of good scientific methodological quality with low risk of bias. Both studies explained the spirometry results in terms of ‘lung age’. The follow-up time was one month^14^ and one year^12^, respectively.

The combination of counselling and pharmacotherapy is important in determining quit rates. Counselling can range from a brief offer of advice to a more intensive session offering more extended support, and the effects increase according to the intensity of the counselling and the type of medication used. To further increase smoking cessation rates, feedback of physical measurements or potential future effects of smoking (exhaled carbon monoxide, lung function or genetic susceptibility to lung cancer) has been used. However, demonstrated evidence of these types of feedback is limited. In the Cochrane systematic review by Bize et al.^21^ it was concluded that one^12^ of three studies evaluating the effect of spirometry showed a significant effect. This is in agreement with our review, showing significant effects found in this study^12^ and in the later study by Ojedokun et al.^14^.

A simple brief intervention such as spirometry is not necessarily expected to result in increases in cessation when delivered in isolation. The use of spirometry and lung age has been identified as a potential enhancement to delivering brief advice and is hypothesized to work by increasing readiness to quit and willingness to make a quit attempt.

Cultural differences, attitudes and exposure to smoking may differ among the different countries as well as regularity of lung function measurements in clinical practice, which could affect generalizability. Four of the studies were European^10-12,14^. The subjects in the included studies were smokers who were recruited from the general population or from various medical institutions. The studies included both men and women, though the precise proportions were often not specified in the studied groups. One weakness concerning transferability is that the definition of being a smoker was often not precisely specified. Another shortcoming may be the difficulty of comparing the intervention with conventional smoking cessation counselling, since it may be hard to establish what to include as general information about an individual’s health status or possible lung disease.

The number of participants in the studies varied (n = 45–567). The size of the random sample had been calculated prior to the commencement of the studies that showed statistically significant improvement^12,14^. One additional study^10^ included such calculations, but the other studies did not calculate the size of the random sample, and the samples were relatively small, which is associated with a risk of low power that can result in missing smaller effects on smoking cessation rates.

In all the studies, the intervention involved informing the subjects about their FEV1, FEV1/FVC or lung age with or without an explanatory graphic display according to Fletcher et al.^3^. Showing smokers this graphic with a comparison to the individual’s chronological age may be a simple way to explain the concept of lung age. The best way to provide this feedback to the smoker remains unclear, and in the clinical setting this process must be customized to the individual. As yet, there is no study on spirometry as a tool to generate interest in smoking cessation and to initiate discussion on the subject, this might prove to be complicated but could give valuable information of spirometry as a possible motivational tool. There is evidence to support the notion that more intensive interventions yield better results than the minimal input of counselling by doctors^22^. It also remains unclear who should provide the information. Possible differences in the results from the included trials could depend on how the information is given and by whom. In the two studies that showed a benefit from the intervention in addition to smoking cessation counselling, doctors delivered the information^12,14^. Nurses, biomedical analysts and pulmonary function therapists provided the information in some studies. It is possible that contributions from several team members could further improve motivation to quit smoking and spirometry might eventually be useful as part of more comprehensive smoking cessation interventions. Repetition of information might be valuable as well as accessible information such as graphic presentations. Spirometry and lung age have been identified as a potential enhancement to delivering brief advice and is hypothesized to work by increasing patient motivation and readiness to quit, which in turn could increase the likelihood for a patient to make a quit attempt and potentially increase the quit rate. Information of spirometry results may stimulate the idea of becoming smoke-free, and the addition of behavioral change support or pharmacology might be crucial to success. Regarding long-term effects, the scientific basis is currently insufficient.

Spirometry should of course be conducted correctly using suitable, reliable equipment, and the information should be appropriately provided to smokers. However, no study has addressed these issues. Choice of spirometry equipment varied among the different studies, which reflects the large selection in the market. The Ojedokun et al.^14^ study used a simple handheld spirometer, COPD-6^TM^ (Vitalograph, Ennis, Ireland), which is user-friendly and less resource-intensive than conventional spirometry. The Swedish National Board of Health and Welfare’s 2015 National Guidelines for Asthma and COPD highly prioritize this type of FEV1/FEV6 measurement in the initial workup of pulmonary obstructive disease in patients who smoke or have smoked, where COPD is suspected^23^. The COPD-6^TM^ screener is currently being used to some extent in primary care in Sweden. Another option in the market is the PiKo-6 lung health monitor (NSpire Health Ltd, Longmont, USA). The Parkes et al.^12^ study used a simple portable MicroLab 3500 spirometer (Micro Medical, Chatham, Kent, UK).

Costs related to the intervention were briefly discussed in two studies. Cost per successful smoking cessation for one patient was estimated at €337 in the Parkes et al.^12^ study. According to Ojedokun et al.^14^, the addition of 1 to 2 minutes of treatment time could be converted into a cost-effective and clinically-effective intervention that can easily be incorporated into routine medical practice. According to the National Board of Health and Welfare^24^ there is evidence to support the assertion that: ‘expert individual counselling to adults who smoke daily entails low costs per quality-adjusted life year and life-year gained compared with conventional or no treatment at all’. The studies referred to in the national guidelines^24^ show a cost in the range €590 – €1240 per quality-adjusted life year (QALY) compared with the alternative^25,26^. Other studies reported the effect in terms of life-years gained and arrived at a cost of €510 – €1020 per life-year gained^27,28^.

No study is currently available that has explored whether the added time required to perform spirometry followed by feedback to the patient, in addition to expert counselling, impacts cost-effectiveness. As a comparison, it can be mentioned that, for common diagnoses, the Swedish Dental and Pharmaceutical Benefits Agency allows the cost per QALY to amount to about €50000 in order to be covered by the benefit. This can be viewed as the society’s willingness to pay and, in this perspective, refraining from expanding smoking cessation interventions would entail a socioeconomic loss and, of course, loss of health for patients. No study has reported whether any negative consequences arose from the intervention, which could be important from an ethical perspective. However, that is unlikely since spirometry is a non-invasive method without serious side effects. In addition to a likely reinforcement of the smoking cessation process, spirometry may give other advantages such as diagnosing COPD or diseases with restrictive lung function, that is, it may indicate that the patient is ill.

There are five published study protocols for RCTs evaluating the use of spirometry as a strategy for smoking cessation^16-20^. Results from these studies can be expected to affect the current state of scientific knowledge within a few years.

## CONCLUSIONS

This systematic review shows that two RCTs with low risk for bias demonstrated a benefit from including feedback of spirometry results, expressed as lung age, in smoking cessation counselling. There is currently only limited evidence to support the use of feedback from spirometry results in addition to smoking cessation counselling with the aim of increasing smoking quit rates.

## CONFLICTS OF INTEREST

The authors have completed and submitted the ICMJE Form for Disclosure of Potential Conflicts of Interest and none was reported.
