# Supplementary material for: Spirometry to increase smoking cessation rate: A systematic review
Source: Tob Induc Dis. 2019 Apr 17;17:31. doi: 10.18332/tid/106090 (PMC6662778; doi:10.18332/tid/106090)
Supplement: Supplementary file 1 [file TID-17-31-s1.pdf]

Appendix 1. Search strategies

Pubmed 170327

Söktermer			Antal träffar
Population: Personer som deltar i rökavvänjningsprogram			
	1.	smoking cessation[MeSH Terms]	23714
	2.	smok*[Title/Abstract]) OR "smoking cessation"	233951
	3.	1. OR 2.	233951
	4.	intervention[Title/Abstract] OR program*[Title/Abstract] OR support[Title/Abstract] OR cessation[Title/Abstract] OR stop[Title/Abstract])	1852223
Intervention: Mätning av lungfunktion			
	5.	Spirometry[MeSH Terms] OR Respiratory Function Tests[MeSH Terms]	212805
	6.	respiratory function test*[Title/Abstract] OR fev1[Title/Abstract] OR forced expiratory volume[Title/Abstract] OR "lung age"[Title/Abstract] OR copd-6[Title/Abstract] OR copd6[Title/Abstract] OR vitalograph[Title/Abstract] OR spirometr*[Title/Abstract])	42698
	7.	5. OR 6.	224912
Kombinerade set			
	8.	3. AND 4. AND 7.	1711
Limits Studietyp: clinical trial, rct, metaanalys, systematisk översikt			
	9.	8.	323
	10.	Efter dubblettborttagning	316

Medline 170327

Söktermer			Antal träffar
Population: Personer som deltar i rökavvänjningsprogram			
	1.	AB ((smoke OR smoking) N3 stop or quit or cessation) OR TI ((smoke OR smoking) N3 stop or quit or cessation)	67626
Intervention: Mätning av lungfunktion			
	2.	AB (fev1 OR "lung age" OR "forced expiratory volume" OR "Respiratory Function Test* OR copd-6 OR copd6 OR Vitalograph OR spirometr*) OR TI (fev1 OR "lung age" OR "forced expiratory volume" OR "Respiratory Function Test* OR copd-6 OR copd6 OR Vitalograph OR spirometr*)	50140
Kombinerade set			
	3.	1. AND 2.	904
Limits Studietyp: clinical trial, rct, metaanalys, systematisk översikt			
	4.	3.	191
	5.	Efter dubblettborttagning	58

Cochrane 170327

Söktermer			Antal träffar
Population: Personer som deltar i rökavvänjningsprogram			
	1.	MeSH descriptor: [Smoking Cessation]	3703
	2.	smoke or smoking:ti,ab,kw and quit or stop or cessation:ti,ab,kw	16590
	3.	1. OR 2.	16590
Intervention: Mätning av lungfunktion			
	4.	fev1 or lung age or forced expiratory volume or Respiratory Function Test or copd-6 or copd6 or Vitalograph or spirometry:ti,ab,kw	33164
Kombinerade set			
	5.	3. AND 4.	422
Limits Studietyp: rct, metaanalys, systematisk översikt			
	6.	5.	414
	5.	Efter dubblettborttagning	243

Cinahl 170327

Söktermer			Antal träffar
Population: Personer som deltar i rökavvänjningsprogram			
	1.	AB ((smoke OR smoking) N3 stop or quit or cessation) OR TI ((smoke OR smoking) N3 stop or quit or cessation) OR MH "Smoking Cessation" OR MH "Smoking Cessation Programs	25675
Intervention: Mätning av lungfunktion			
	2.	AB (fev1 OR "lung age" OR "forced expiratory volume" OR "Respiratory Function Test* OR copd-6 OR copd6 OR Vitalograph OR spirometr*) OR TI (fev1 OR "lung age" OR "forced expiratory volume" OR "Respiratory Function Test* OR copd-6 OR copd6 OR Vitalograph OR spirometr*)	43395
Kombinerade set			
	3.	1. AND 2.	552
Limits Studietyp: clinical trial, rct, metaanalys, systematisk översikt			
	4.	3.	136
	5.	Efter dubblettborttagning	54

Embase 170314:

Söktermer			Antal träffar
Population: Personer som deltar i rökavvänjningsprogram			
	1.	smoking cessation/ or smoking cessation program/	51533
	2.	"smok*".ab,ti.	323781
	3.	(cessation or stop* or quit*).ab,ti.	360566
	4.	2 adj3 3.ab,ti.	35908
	5.	1 or 4	58569
Intervention: Olika typer av lungfunktionstest			
	6.	lung function test/ or spirometry/	78610
	7.	("forced expiratory volume" or "lung function test*" or "respiratory test*" or fev1 or "pulmonary function test*" or "ventilation test*" or "lung age" or "copd 6" or copd6 or vitalograph or spirometr* or "breath measurement").ab,ti.	81546
	8.	6 or 7	119315
Kombinerade set			
	9.	5 and 8	2129
Limit: tar bort böcker, kapitel, konferensabstract och konferenssammanfattningar			
	10.	limit 9 to (book or chapter or conference abstract or "conference review")	473
	11.	9 not 10	1656
Studietyp: clinical trial, metaanalys, systematisk översikt			
	12.	exp clinical trial/	1336749
	13.	11 and 12	254
	14.	limit 9 to (meta analysis or "systematic review")	49
	15.	("meta stud*" or "meta analysis" or "systematic review" or trial or random*).ab,ti.	1607602
	16.	11 and 15	222
	17.	13 or 14 or 16	377
		Efter dubblettborttagning	274

- Ang. rad 6 – I Embase är sökning endast gjord på ämnesorden ”Lung Function Test” och ”Spirometry” efter kontakt med Örebro.

Amed 170314:

Söktermer			Antal träffar
Population: Personer som deltar i rökavvänjningsprogram			
	1.	smoking cessation/	199
	2.	"smok*".ab,ti.	1203
	3.	(cessation or stop* or quit*).ab,ti.	1449
	4.	2 adj3 2.ab,ti.	271
	5.	1 or 4	326
Intervention: Olika typer av lungfunktionstest			
	6.	exp respiratory function tests/	1404
	7.	("forced expiratory volume" or "lung function test*" or "respiratory test*" or fev1 or "pulmonary function test*" or "ventilation test*" or "lung age" or "copd 6" or copd6 or vitalograph or spirometr* or "breath measurement").ab,ti.	846
	8.	6 or 7	1964
Kombinerade set			
	9.	5 and 8	11
Studietyp: clinical trial, metaanalys, systematisk översikt			
	10.	("meta stud*" or "meta analysis" or "systematic review" or trial or random*).ab,ti.	20858
	11.	9 and 10	1

PsycInfo 170314:

Söktermer			Antal träffar
Population: Personer som deltar i rökavvänjningsprogram			
	1.	smoking cessation/	11270
	2.	"smok*".ab,ti.	46355
	3.	(cessation or stop* or quit*).ab,ti.	70549
	4.	2 and 3	14294
	5.	1 or 4	16427
Intervention: Olika typer av lungfunktionstest			
	6.	("forced expiratory volume" or "lung function test*" or "respiratory test*" or fev1 or "pulmonary function test*" or "ventilation test*" or "lung age" or "copd 6" or copd6 or vitalograph or spirometr* or "breath measurement").ab,ti.	834
Kombinerade set			
	7.	5 and 6	30
Studietyp: clinical trial, metaanalys, systematisk översikt, metasyntes			
	8.	limit 7 to ("0300 clinical trial" or "0830 systematic review" or 1200 meta analysis or 1300 metasynthesis)	2
		Efter dubblettborttagning	0

© 2019 Westerdahl E.
